# Modeling appendicular skeletal cartilage development with modified high-density micromass cultures of adult human bone marrow-derived mesenchymal progenitor cells

**DOI:** 10.1186/s13287-019-1505-5

**Published:** 2019-12-16

**Authors:** Alessandro Pirosa, Karen L. Clark, Jian Tan, Shuting Yu, Yuanheng Yang, Rocky S. Tuan, Peter G. Alexander

**Affiliations:** 1 Center for Cellular and Molecular Engineering, Department of Orthopaedic Surgery, University of Pittsburgh School of Medicine, 450 Technology Drive, Pittsburgh, PA 15219 USA; 2 Institute for Tissue Engineering and Regenerative Medicine, The Chinese University of Hong Kong, Shatin, Hong Kong SAR China

**Keywords:** Human bone marrow-derived mesenchymal progenitor cells, High-density micromass culture, Organotypic culture model, Chondrogenesis, Methacrylated gelatin, Collagen type II promoter-reporter, Non-invasive analysis

## Abstract

**Background:**

Animal cell-based systems have been critical tools in understanding tissue development and physiology, but they are less successful in more practical tasks, such as predicting human toxicity to pharmacological or environmental factors, in which the congruence between in vitro and clinical outcomes lies on average between 50 and 60%. Emblematic of this problem is the high-density micromass culture of embryonic limb bud mesenchymal cells, derived from chick, mouse, or rat. While estimated predictive value of this model system in toxicological studies is relatively high, important failures prevent its use by international regulatory agencies for toxicity testing and policy development. A likely underlying reason for the poor predictive capacity of animal-based culture models is the small but significant physiological differences between species. This deficiency has inspired investigators to develop more organotypic, 3-dimensional culture system using human cells to model normal tissue development and physiology and assess pharmacological and environmental toxicity.

**Methods:**

We have developed a modified, miniaturized micromass culture model using adult human bone marrow-derived mesenchymal progenitor cells (hBM-MPCs) that is amenable to moderate throughput and high content analysis to study chondrogenesis. The number of cells per culture was reduced, and a methacrylated gelatin (gelMA) overlay was incorporated to normalize the morphology of the cultures.

**Results:**

These modified human cell-based micromass cultures demonstrated robust chondrogenesis, indicated by increased Alcian blue staining and immunodetectable production of collagen type II and aggrecan, and stage-specific chondrogenic gene expression. In addition, in cultures of hBM-MPCs transduced with a lentiviral collagen type II promoter-driven GFP reporter construct, levels of GFP reporter activity correlated well with changes in endogenous collagen type II transcript levels, indicating the feasibility of non-invasive monitoring of chondrogenesis.

**Conclusions:**

The modified hBM-MPC micromass culture system described here represents a reproducible and controlled model for analyzing mechanisms of human skeletal development that may later be applied to pharmacological and environmental toxicity studies.

## Background

Congenital limb defects afflict 3–6 in 10,000 live births [1, 2]. While not life-threatening, these defects exact a large economic and societal burden on affected individuals and health care systems [3, 4]. Over 50% of these abnormalities have an unknown etiology and are classified as multifactorial, caused by a combination of genetic susceptibility and one or more environmental exposures [5]. Humans are exposed to thousands of chemicals with unknown and untested biological consequences, leaving open the possibility that environmental factors could play a very significant role in the development of skeletal defects [6–8]. To assess the environmental component of limb defect etiology, thousands of chemicals will need to be tested. According to current international guidelines, developmental toxicity testing involves exposure of pregnant animals, predominantly rats and rabbits, and subsequent assessment of toxic effects in dams and their fetuses [9]. These studies are costly and poorly predictive of human embryotoxicity due to the physiological differences between animal models and humans [10–12]. Thus, there is a high demand for less costly alternative in vitro cell culture models that allow for direct mechanistic analysis of target cell populations [10, 13]. In vitro techniques for the study of embryonic limb skeletogenesis have been available for some time. One historically informative model is the high-density micromass culture of embryonic limb bud mesenchyme [14–16]. The micromass culture is a convenient model for the observation and analysis of processes involved in the differentiation of limb cartilage anlagen in situ. Unstimulated, the embryonic limb mesenchymal cells undergo an early proliferative and condensation phase that gives rise to cartilaginous aggregates or nodules, mimicking the phenomenon that occurs during embryonic limb development in vivo [14, 17–20]. Further stimulation with triiodothyronine induces hypertrophy in these cultures that calcify under appropriate culture conditions [21, 22]. These cultures have even been used to analyze early events in joint formation, i.e., interzone formation and downregulation of chondrogenesis [21, 22]. The flexibility and spontaneity of the culture reflects the endogenous, specified state or fate of this cell population. In fact, the rat embryonic limb bud mesenchyme micromass culture is one of a few validated in vitro models for developmental toxicity/embryotoxicity testing [16], recording an 84% success rate in anticipating teratological effects of tested environmental chemicals [16, 23, 24]. However, overall, the congruence between animal models and clinical outcomes stands at approximately 50% [25]. Thus, regulatory agencies around the globe are funding the development of human cell-based in vitro tissue models for developmental toxicity testing, made possible by advancements and convergence of tissue engineering, stem cell technologies, microfluidic-enabled culture platforms, and high throughput/high content analysis.

Mesenchymal progenitor cells (MPCs) are relatively quiescent undifferentiated cells residing within every tissue of the human body that, when activated by injury, orchestrate and participate in the healing and regenerative response [26–28]. MPCs are easily isolated through selective adhesion and/or gradient centrifugation in relatively high numbers, e.g., 1:10,000 nucleated cells in bone marrow and up to 2 to 5% in the stromal vascular fraction of adipose, and may be expanded through 10–12 cell divisions (usually to passage 5 during in vitro culture) before losing potency [29, 30]. Thus, depending on tissue source and volume of isolate, 10^6^ to 10^7^ of cells with progenitor cell characteristics, i.e., rapid plastic adherence, tri-lineage differentiation, and specific surface antigen expression patterns (positive for CD73, CD90, and CD105 while negative for CD31, CD34, and CD 45, among others [31]), may be isolated for tissue engineering and regenerative processes or in vitro individualized therapeutic or toxicity testing. In orthopedic research and tissue engineering, these cells are used to generate skeletal tissues including bone, cartilage, tendon, and ligament, depending on the scaffolds and growth factors used in the process [28]. There are currently more than 80 clinical trials for the use of progenitor cells in musculoskeletal tissue regeneration and tissue engineering, 45 of which are in phase II/III (www.clinicaltrials.gov, see also [32, 33]). MPCs are able to undergo chondrogenic differentiation reminiscent of hyaline cartilage formation in adults and have been shown to recapitulate the processes and associated molecular regulations of the embryonic endochondral ossification, spatially and temporally. In particular, these morphogenic processes include cellular condensation and chondrocyte hypertrophy, Indian Hedgehog signaling, metalloproteinase-mediated remodeling, vascularization, and bone formation [34, 35]. By leveraging the ability of adult MPCs to comply with developmental engineering, we report on modifications of the conventional micromass system for use with adult human bone marrow-derived mesenchymal progenitor cells (hBM-MPCs), applicable in the future for high content, moderate throughput analytic techniques for assessing natural and man-made environmental chemical embryotoxicity.

## Methods

### Materials and reagents

All reagents were purchased from Sigma-Aldrich unless otherwise stated. Methacrylated gelatin (gelMA) was obtained through the reaction between gelatin and methacrylic anhydride (MA) as previously described [36]. The photoinitiator lithium phenyl-2,4,6-trimethylbenzoylphosphinate (LAP) was synthesized as previously described by Fairbanks et al. [37].

### Culture media

hBM-MPC growth medium (GM): Dulbecco’s modified Eagle medium (DMEM) supplemented with 10% fetal bovine serum (FBS) and 2% penicillin/streptomycin/fungizone; hBM-MPC chondrogenic medium (CM): GM without FBS, supplemented with 10 ng/mL TGF-β3, 1% insulin-transferrin-selenium, 50 μM L-ascorbic acid-2 phosphate, 10 nM dexamethasone, and 23 μM proline.

### Cell culture

hBM-MPCs were obtained, with IRB approval of the University of Pittsburgh, from femoral heads of patients who underwent total joint arthroplasty, according to a previously described procedure [38]. hBM-MPCs were cultured as monolayer in GM at 37 °C and 5% CO_2_. GM was changed every 2–3 days until ~ 80–90% confluency. Aliquots of cells were collected before initial plating (p0) for CFU analysis (Additional file 1: Table S1) and at p2 for CFU (Additional file 1: Figure S1), tri-lineage differentiation (Additional file 1: Figure S2; Supplementary Methods), and surface antigen profile (Additional file 1: Figure S3) as previously described [39], with properties summarized in Additional file 1: Table S1. All experiments were performed in triplicates, using cells pooled from 8 different donors (mean age = 54.5 yo, age range = 38–76 years old).

### Development of modified micromass culture

hBM-MPCs were expanded in GM to passage 3, pelleted, and adjusted to a density of 20 × 10^6^ cells/ml. Each high-density micromass was formed by pipetting a 2-μl drop of cell suspension into the center of each well of a 48-well culture plate which was previously texturized and coated with collagen type I (see the “Results” section for details) to optimize cell attachment. Cells were allowed to sediment and adhere to the plate for 30 min, then covered with CM overnight. Subsequently, a 5% gelMA/0.15% LAP (w/v) PBS solution was pipetted on top of the micromass and photocrosslinked for 2 min using a blue light (450–490 nm wavelength) as described previously [40]. The cultures were then cultured in CM for 14 days with medium changed every 2 days. The micromass cultures were collected at days 7, 10, and 14 for subsequent analyses.

### MTS and glucose assays

hBM-MPC viability was analyzed using the CellTiter 96® AQueous One Solution Cell Proliferation Assay (MTS) (Promega) according to the manufacturer’s instructions. Briefly, cells were incubated for 1 h at 37 °C with the MTS tetrazolium salt compound, and A_490_ was measured using a BioTek Synergy HT plate reader system (BioTek). hBM-MPC metabolic activity was assessed using the Glucose Assay kit (Colorimetric/Fluorometric) (ab65333, Abcam) according to the manufacturer’s instructions.

### Lentiviral transduction of hBM-MPCs

hBM-MPCs were transduced with a human collagen type II (*COL2A1*) promoter-driven eGFP reporter using the Lentifect™ Lentivirus system (GeneCopoeia). Cells were first suspended in GM and plated in a 96-well culture plate at a density of 5000 cells per well. After 18–20 h, GM was replaced with the lentiviral suspension (in GM without penicillin/streptomycin/fungizone) at a multiplicity of infection (MOI) of 50 (MOI = number of TU/number of cells per well) in the presence of SureEntry (a transduction enhancing reagent from Qiagen) added at 6 μg/ml. After 24 h of incubation, the lentiviral suspension was replaced with fresh GM. Transduced hBM-MPCs were harvested after 3 days and combined 1:100 with non-transduced cells, which were plated together at a density of 20 × 10^6^ cells/ml and cultured as described above.

### Fluorescence intensity quantification

ImageJ software (NIH) was used to calculate the corrected total cell fluorescence (CTCF) in images (× 10 magnification) obtained by epifluorescence microscopy. The area inside cells and background were highlighted, and “area”, “mean grey value,” and “integrated density” were recorded. CTCF was calculated as [integrated density − (area of selected cell × mean fluorescence of background readings)].

### Histology and immunohistochemistry

Samples were fixed in 4% paraformaldehyde in PBS, paraffin-embedded following standard procedures, and sectioned at 6-μm thickness. For histology, sections were rehydrated and stained with Alcian blue to detect sulfated glycosaminoglycans and hematoxylin/eosin (H&E) to study cell morphology. For immunohistochemistry (IHC), sections were processed for enzymatic antigen retrieval involving incubation in chondroitinase/hyaluronidase in 0.02% bovine serum albumin (BSA) solution in phosphate-buffered saline (PBS) for 30 min at 37 °C. Subsequently, samples were pre-incubated for 10 min with 3% H_2_O_2_ in methanol solution to quench endogenous peroxidase activity. Nonspecific binding was then suppressed with 1% horse serum (Vector Labs) in PBS for 45 min. Following antigen retrieval and blocking, sections were incubated overnight at 4 °C with primary antibodies against human collagen type II (Abcam, ab34712) or against aggrecan (Abcam, ab3778) at a dilution of 1:400 and 1:100, respectively, followed by 30-min incubation with biotinylated secondary antibody (Vector Labs). Immunostaining was detected using horseradish peroxidase (HRP)-conjugated streptavidin and Vector® NovaRED™ peroxidase substrate, with hematoxylin (Vector Labs) as counterstain. After staining, both histology and IHC slides were dehydrated, mounted, coverslipped, and imaged with a Nikon Eclipse E800 microscope (Nikon Instrument).

### Scanning electron microscopy

Micromasses were fixed in 3% glutaraldehyde in PBS for 1 h. Samples were then washed three times in PBS, post-fixed for 1 h in aqueous 1% osmium tetroxide, and then washed three times in PBS. Samples were dehydrated through a graded ethanol series (30–100%) and further dehydrated by three additional 15-min washes with absolute ethanol. Next, the samples were washed in hexamethyldisilizane (HMDS) for 30 min and then removed to air dry. Samples were then mounted onto aluminum stubs and sputter coated with 5 nm gold/palladium (Cressington Sputter Coater Auto 108, Cressington, Watford, UK). Images were acquired using a JEOL JSM-6335F scanning electron microscope (SEM) (Peabody, MA) at 3 kV.

### Gene expression analysis

Total RNA was extracted using Trizol (Invitrogen) and purified with the RNeasy Plus mini kit (Qiagen). cDNA was reverse transcribed using the SuperScript IV kit (Invitrogen). Quantitative real-time PCR was performed using a StepOnePlus thermocycler (Applied Biosystems) and SYBR Green Reaction Mix (Applied Biosystems). Expression of aggrecan (*ACAN*) and collagen type II (*COL2A1*) was quantified to analyze chondrogenic differentiation. 18S rRNA levels were used as endogenous control, and gene expression fold changes were calculated by the comparative cycle threshold (CT) method, using expression levels of undifferentiated cells as reference for the 2^−ΔΔCT^ calculation (Table 1).

**Table 1:** Primer sequences for qRT-PCR analysis of gene expression

	Forward 5′-3′	Reverse 5′-3′
*18S rRNA*	GTA ACC CGT TGA ACC CCA TT	CCA TCC AAT CGG TAG TAG CG
*ACAN*	AGT CAC ACC TGA GCA GCA TC	AGT TCT CAA ATT GCA TGG GGT GTC
*COL2A1*	GGA TGG CTG CAC GAA ACA TAC CGG	CAA GAA GCA GAC CGG CCC TAT G

### Statistical analysis

Each sample was assayed in triplicate, and the quantitative data were reported as mean ± SD. Statistical analyses were performed using GraphPad Prism 7 (GraphPad Software Inc.). Student’s *t* test and one-way ANOVA/post hoc Tukey test were used to compare two or more independent groups, respectively. Statistical tests were two-tailed, and significance was set at *p* ≤ 0.05.

## Results

### Development of a morphologically consistent hBM-MPC-based chondrogenic micromass culture

Chondrogenic differentiation of adult hBM-MPCs has been commonly assayed in high-density, non-adherent pellet cultures that exhibit robust chondrogenesis, but are not amenable for routine, non-invasive quantitative analysis, in particular histological and microscopic examination. In comparison, planar cultures, such as the high-density, substrate-adherent micromass system first developed and used for embryonic limb bud mesenchymal cells, allow for observation and analysis of temporal and spatial cell activity throughout the culture period. Micromass cultures are generally characterized on the basis of the high plating cell density, typically 20 × 10^6^ cells/ml, which is reminiscent of the cell density found in the embryonic limb bud at the pre-condensation step in vivo (chicken: Hamilton Hamburger stage 20–24; and mouse: embryonic day 11.5–12.5) [41]. In adapting the micromass culture system for the use of adult human MPCs, several modifications were necessary. As hBM-MPCs generally show diminished chondrogenic differentiation potency after passage 3 [42], we calculated that micromass culture sizes of 40,000 cells or less would allow up to 500 cultures to be conveniently set up with passage 3 cells, under routine hBM-MPC harvesting and culture conditions. By keeping the cell density at 20 × 10^6^ cells/ml, a first consideration was thus reduction in culture size, i.e., from 10 μL with 200,000 cells down to 2 μL with 40,000 cells. Unfortunately, it was found that 10 μL micromass cultures (of 200,000 cells) maintained in CM with 10 ng/ml TGFβ3 adhered and differentiated on standard, tissue culture-treated polystyrene surfaces over a 21-day period (data not shown), while 2-μL cultures (of 40,000 cells) consistently detached from the culture surface within days after culture to form an irregular pellet culture (Additional file 1: Figure S4). This behavior could be related to the higher mechanical strength of the larger micromass, with larger contact interface, that could increase its stability and attachment to the plate.

**Fig. 1:**
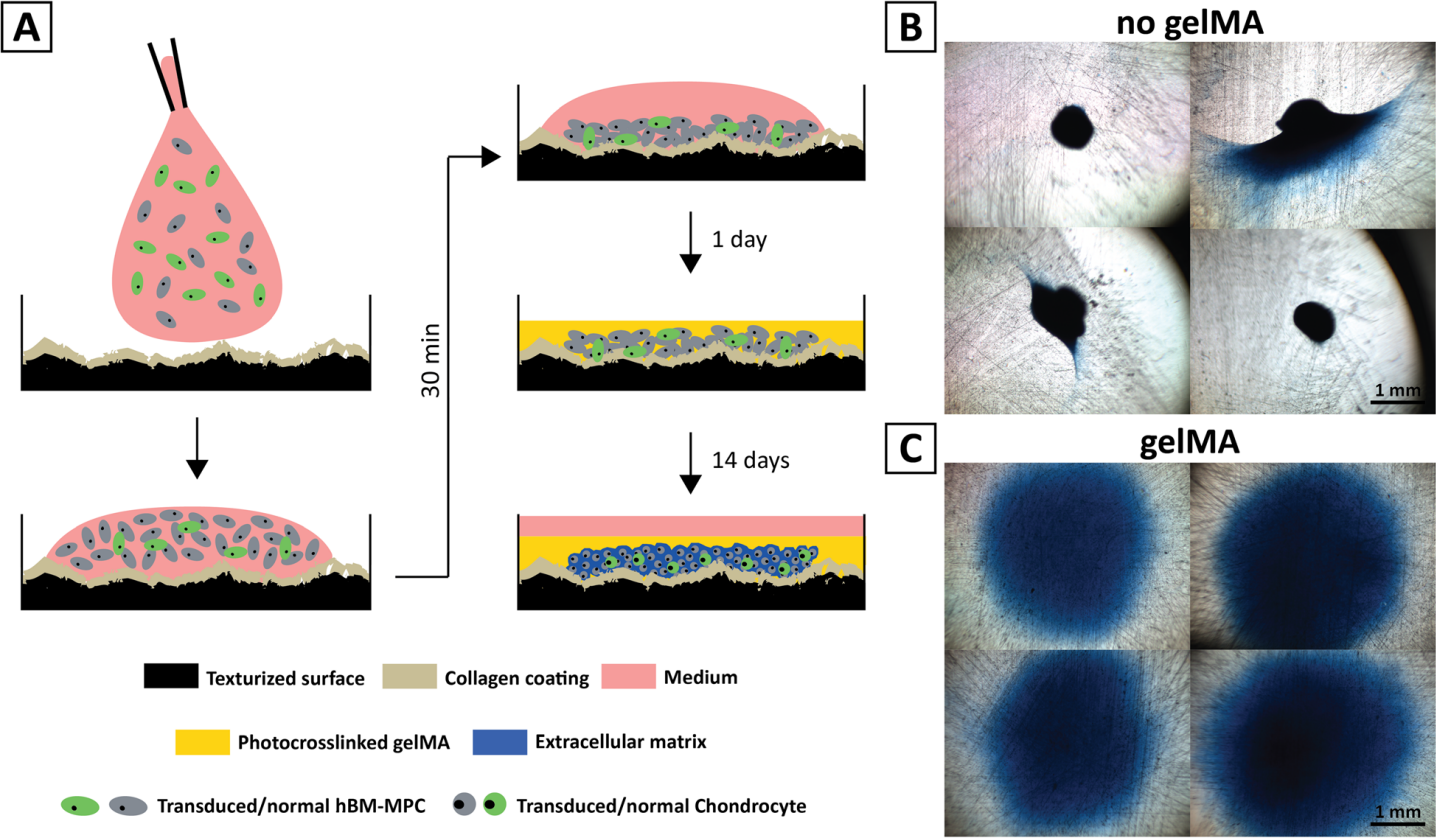
Schematic of chondrogenic organotypic culture model. **a** Preparation of gelMA overlay, high-density hBM-MPC micromass cultures: A 2-μL droplet of 20 × 106 cells/ml hBM-MPC s was placed on the texturized and collagen-coated surface cultures. After 30 min of cell attachment, a 100-μL aliquot of photocrosslinked gelMA (5%) was placed on top of the culture, which kept the engineered tissue flat during the 14-day experiment. In some experiments, 1/100 of the cells in the micromass culture consisted of hBM-MPCs transduced with a lentiviral *COL2A1* promoter-driven GFP reporter, applicable for non-invasive assessment of chondrogenesis. **b**, **c** Alcian blue staining of day 14 hBM-MPC micromass cultures (**b**) with and (**c**) without gelMA overlay, showing differences in culture morphology and GAG production

Substrate coating with extracellular matrix (ECM) is frequently used to promote cell-substrate adhesion, with collagen type I known to promote MPC adhesion [43]. To improve the attachment of hBM-MPCs seeded as a 2-μL micromass, 48-well plates were first coated with collagen type I by adding 200 μL of 1 mg/ml collagen type I solution (PureCol EZ-Gel) to individual wells for 2 h at room temperature, followed by rinsing once with PBS and air-drying. However, despite initial improved adhesion, the majority of the 2-μL cultures still detached from the substrate surface over the 14-day duration of the experiment (Additional file 1: Figure S4). We next investigated texturizing the surface to enhance adhesion. This was achieved by abrasion of the bottom interior surface of the wells of the polystyrene 48-well cultures using 800-grit sandpaper applied in 45° increments. Cells were then applied to the texturized surfaces with or without additional collagen type I coating (as described above). The results showed that texturization combined with collagen coating resulted in micromass cultures that remained attached throughout the 14-day period of differentiation, but the cultures often assumed irregular morphologies not optimal for reproducible, quantitative analysis (Fig. 1b).

We speculated that this was due to lack of control over the 3-dimensional spreading of the seeded cell suspension and that this could be limited or corrected with a hydrogel overlay. Such a construct would have a stable, uniform shape, without curling of the edges, while the hydrogel could also permit nutrient diffusion to and metabolite release from the cells. To test this idea, we chose to use a methacrylated gelatin hydrogel (gelMA) that has tunable mechanical properties and is permissive to chondrogenesis [36, 40, 44]. Cultures were prepared as outlined in Fig. 1a, seeded upon texturized, collagen type I coated surfaces of 48-well plates and maintained in CM containing TGFβ3. The overlay of 5% gelMA resulted in the maintenance of a hBM-MPC micromass with uniform shaped morphology through the duration of the 14-day experiment (Fig. 1c).

Preliminary analysis of hBM-MPC chondrogenic differentiation in these 2-μL micromass cultures revealed enhanced differentiation with the gelMA overlay, suggesting the positive influence of a regular-shaped culture and perhaps additional microenvironmental cues from the ECM (Additional file 1: Figure S5). Taken together, these modifications to the standard micromass culture technique yielded improved, reproducible cultures that presented a larger surface for nutrient, cytokine, and metabolite movement, and lower and uniform cell density for easy microscopic observation as well as biochemical analysis of chondrogenesis (Fig. 1c).

### Chondrogenic differentiation in hBM-MPC-based gelMA overlay micromass cultures

The gelMA overlay micromass cultures were maintained in CM containing TGFβ3, and chondrogenesis was monitored throughout the 14-day period.

#### Morphology

**Fig. 2:**
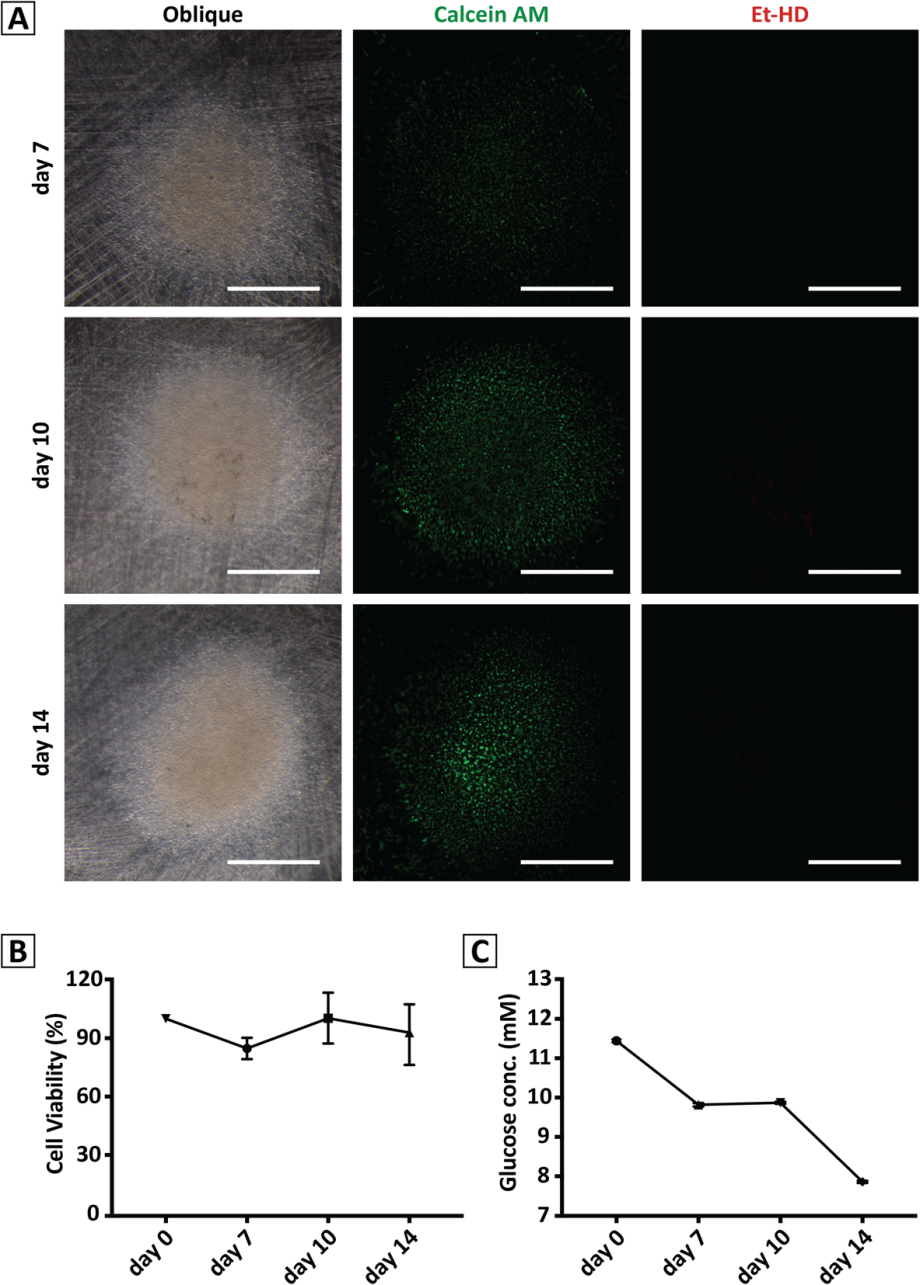
Cytoviability and metabolic characteristics of the gelMA overlay hBM-MPC micromass cultures during TGFβ3-induced chondrogenesis. **a** Oblique bright-field and epifluorescence imaging of micromass cultures on days 7, 10, and 14. Under oblique bright-field microscopy, the matrix rich core of the culture appeared as a yellow field. Under epifluorescence microscopy, Live/Dead™ staining of the same cultures revealed that most of the cells were alive (Calcein: green), with very few dead cells present (Et-HD: red). Scale bar = 1 mm. **b** MTS assay. No significant changes in cell proliferation were seen over the 14-day culture period. **c** Medium glucose concentration analysis. The results showed that glucose consumption by the micromass cultures increased over culture time

Oblique bright-field imaging on an SZX16 stereomicroscope revealed a culture characterized by a dense core surrounded by a monolayer of cells. In addition, as chondrogenesis proceeded, the cells in the core showed a spherical morphology indicative of high matrix production characteristic of chondrocytes, while those located at the edge of the culture were elongated and mesenchymal in shape. Analysis of cell viability in these cultures (Live/Dead™, Life Sciences) revealed that almost all cells in the micromass were alive (green), not dead (red) (Fig. 2a).

#### Biochemical analysis

Data from MTS analysis (Fig. 2b) supported high cell viability, with no decrease of cell number throughout the culture period, while medium glucose assay (Fig. 2c) showed a progressive increase of glucose consumption by cells. These cell behaviors correlated with the chondrogenic nature of the culture, namely that hBM-MPC proliferation was halted while differentiating towards the chondrogenic phenotype, while active ECM biosynthesis resulted in elevated metabolic activity [45, 46].

#### Histology

**Fig. 3:**
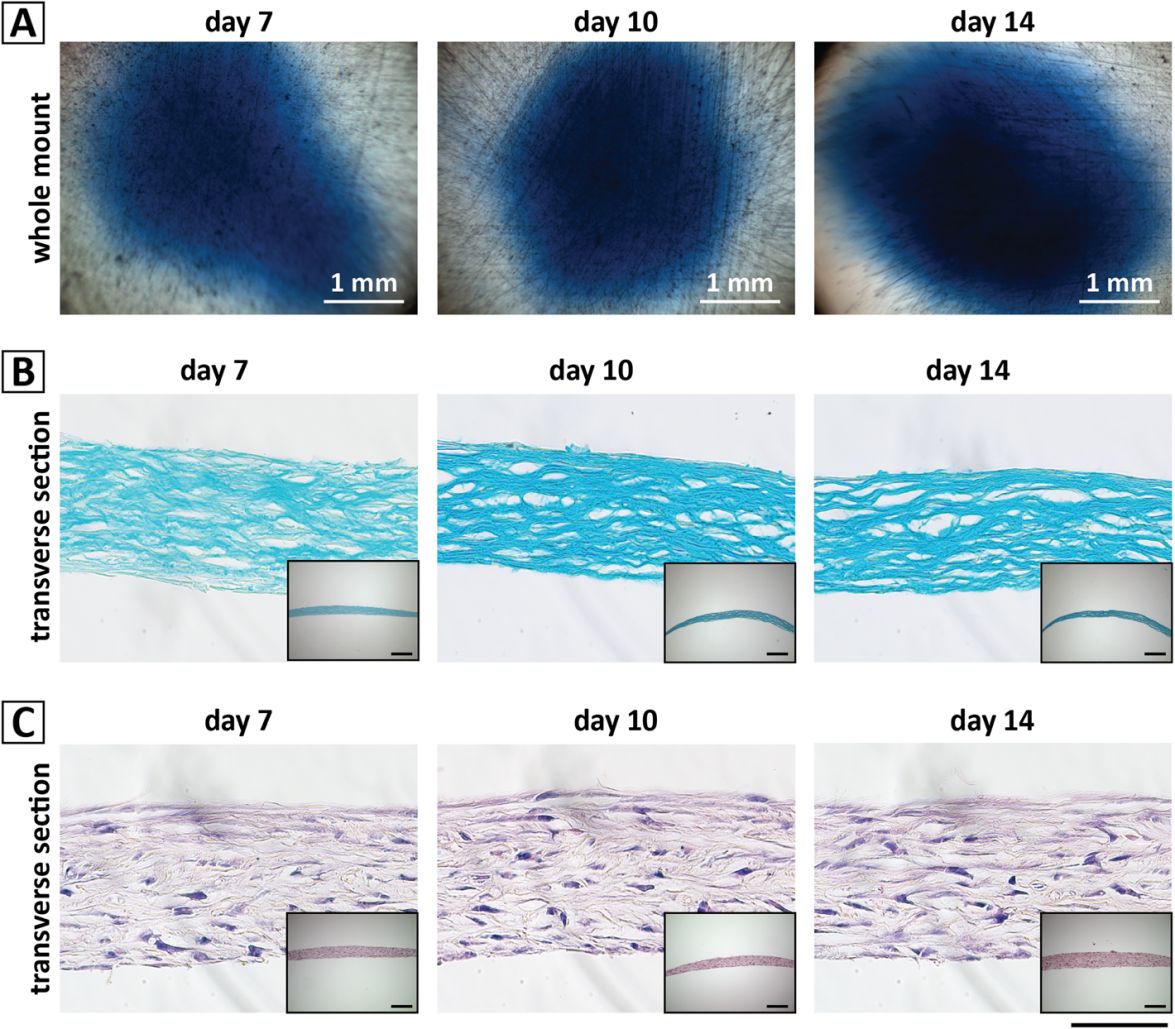
Histological analysis of the hBM-MPC micromass cultures during chondrogenesis. **a** Whole-mount cultures stained with Alcian blue showing cartilage-specific GAG-rich matrix deposition on days 7, 10, and 14 of culture. Scale bar = 1 mm. **b**, **c** Transverse sections of the same cultures stained with **b** Alcian blue, showing abundant, GAG-rich extracellular matrix encasing lacunae, and **c** hematoxylin-eosin, showing uniform cell distribution embedded within abundant extracellular matrix. Scale bar = 50 μm. Inset images depict a low magnification (× 10) image of each culture for reference. Scale bar = 200 μm

The ability of hBM-MPCs to produce a GAG-rich matrix, typical of cartilage, was clearly evident on the basis of dense Alcian blue staining (Fig. 3a) at 7, 10, and 14 days of culture. Photomicrographs of whole-mount and transverse-sectioned micromass cultures stained with Alcian blue revealed a progressive increase in GAG deposition from day 7 to day 14. H&E staining (Fig. 3b) showed the formation of lacuna-like structures typical of hyaline cartilage, with a basophilic matrix surrounding the cells. These histological characteristics suggest the formation of a morphologically relevant, engineered cartilaginous tissue derived from human cells.

#### Scanning electron microscopy

**Fig. 4:**
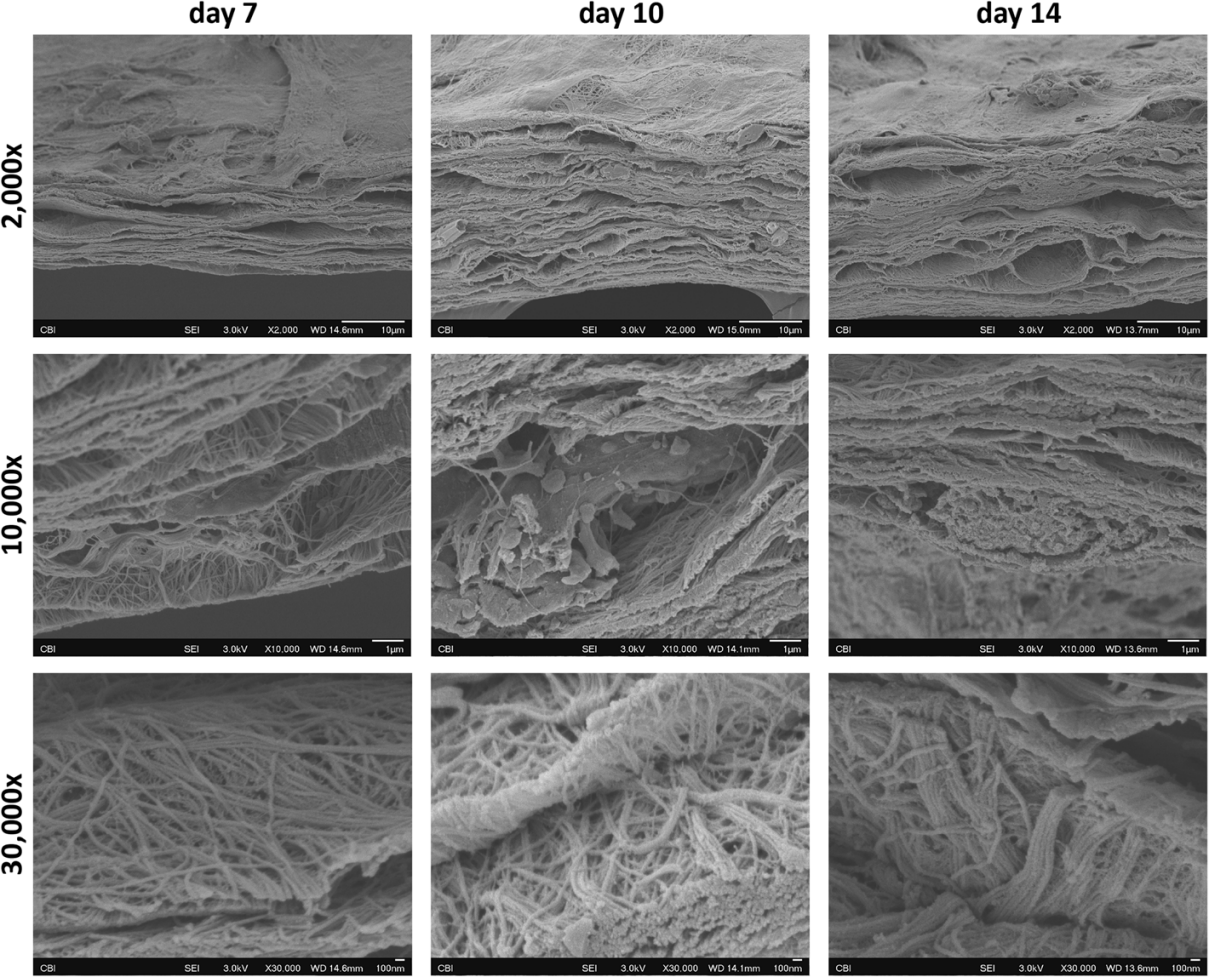
SEM analysis of the hBM-MPC micromass cultures during chondrogenesis. Representative SEM micrographs of the micromasses at 7, 10, and 14 days of culture and at different magnifications, showing the ultrastructure of the cartilaginous matrix, lacunae-like structures, and progressive collagen fibril aggregation with increasing culture time

The ultrastructure of micromasses was further investigated by SEM, revealing the formation of a laminar matrix in which lacuna-like structures harboring the hBM-MPCs were clearly visible (Fig. 4). We also observed accumulation of ECM from day 7 to day 14 reflected in the increasing height of the cultures and, most importantly, the presence of collagen fibers that increased in complexity (fibril number) and organization (i.e., alignment) over the course of the culture period (Fig. 4).

#### Molecular analyses

**Fig. 5:**
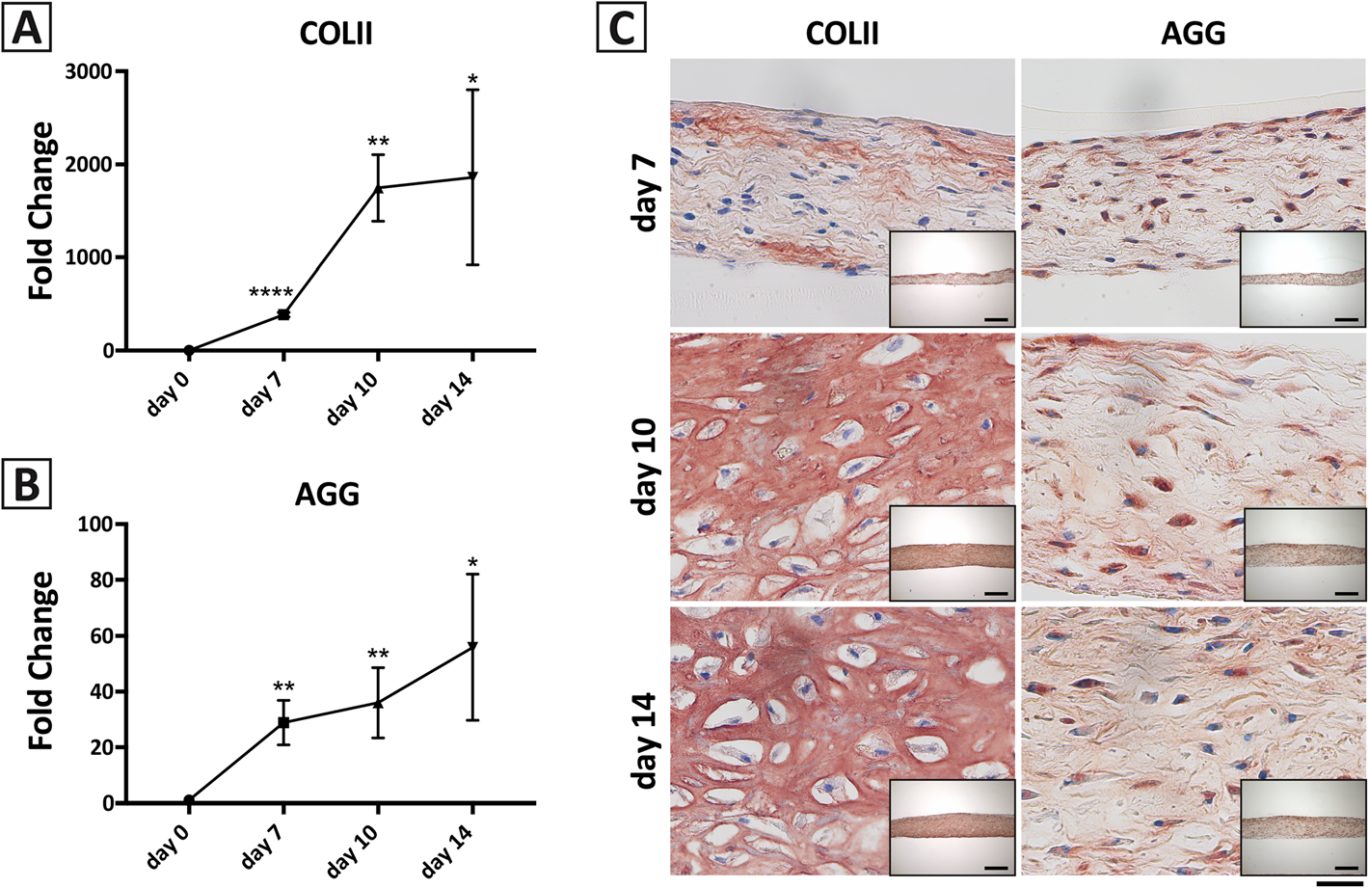
Gene expression and immunohistochemical analyses of hBM-MPC micromass cultures. **a**, **b** qRT-PCR analysis showing time-dependent increase in the expression levels of chondrogenesis-associated genes: **a** collagen type II (*COL2A1*) and **b** aggrecan (*ACAN*), expressed as fold-changes compared to level on day 0 (*p < 0.05; **p < 0.005; ****p < 0.0001). **c** Immunostaining of collagen type II and aggrecan in similar cultures on days 7, 10, and 14 of culture. Scale bar = 50 μm. Inset images depict a low magnification (× 10) image of each culture for reference. Scale bar = 200 μm

The chondrogenic activity of the modified hBM-MPC micromass system was further characterized based on gene expression and immunohistochemical analyses of cartilage-specific markers. qRT-PCR analysis shows a time-dependent increase in *COL2A1* and *ACAN* gene expression over the 14 days of culture, with the greatest rate increase detected between culture days 10 and 14 (Fig. 5a, b). The increased *COL2A1* and *ACAN* transcript levels in cartilage gene expression correlated with the increased immunostaining of matrix deposition of collagen type II and aggrecan proteins in the cultures over time, and morphological maturation of the developing cartilage with collagen type II-rich matrix and lacunae-like structures (Fig. 5c).

Taken together, these findings indicated that the gel overlay hBM-MPC micromass cultures underwent robust chondrogenesis over a 14-day period. The advantages of the modified micromass system were (1) requirement of only a relatively small number of cells and (2) maintenance of a uniform morphology during differentiation using the photocrosslinked gelMA overlay, both of which representing critical features in adapting the cultures to high throughput culture and analysis.

### Non-invasive analysis of chondrogenic differentiation in gelMA overlay hBM-MPC micromass cultures

**Fig. 6:**
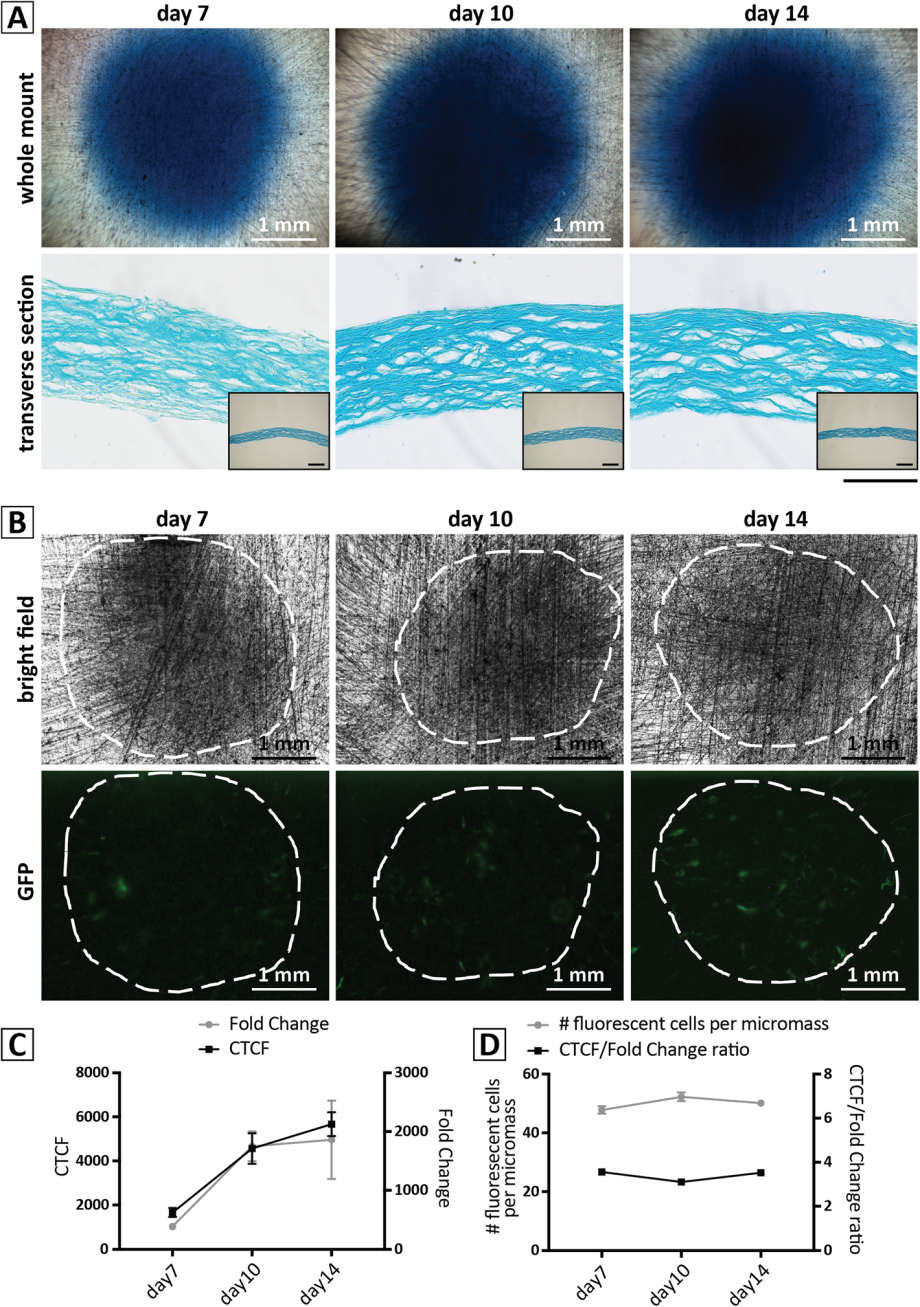
Characterization of chondrogenesis in hBM-MPC cultures containing *COL2A1*-GFP transduced cells as sentinel cells (prepared as described in Fig. 1a and in the text). **a** Alcian blue staining of whole-mount and transverse sectioned samples revealed abundant moderate increases in deposition of cartilage-specific GAG deposition between 7 and 14 days of culture. Scale bar for transverse sections = 50 μm. Inset images depict a low magnification (× 10) image of each culture for reference. Scale bar = 200 μm. **b** Epifluorescence microscopy shows increasing expression of GFP by the transduced sentinel cells, indicating enhanced *COL2A1* promoter activation during chondrogenesis. The white dashed lines indicate the location of the cultures based on the bright-field images of the same cultures. **c** Quantitative analysis of fluorescence (CTCF) in *COL2A1*-GFP transduced cells in the chondrogenic micromass cultures, showing increased fluorescence taking place between 7 and 14 days of culture, in agreement with changes in *COL2A1* gene expression (fold-change) as determined by qRT-PCR in the same cultures. **d** Correspondence between non-invasive, fluorescence-based assay and gene expression analysis. The number of fluorescent cells per micromass did not change while CTCF/gene expression also remained constant throughout the culture period, confirming the correlation between the two outputs

We next investigated the applicability of the modified hBM-MPC micromass system for direct, non-invasive assessment of chondrogenesis, as methods such as RT-PCR and immunohistochemistry were inherently destructive and incompetent with continuous, real-time, and high content/throughput monitoring of differentiation. For this purpose, we adopted the uniform, planar nature of the gelMA hBM-MPC micromass for use with non-invasive imaging techniques, which could be performed in a quantitative manner. As proof-of-concept, we chose to assess the efficacy of a *COL2A1* promoter-GFP lentiviral reporter construct in reporting hBM-MPC chondrogenic differentiation. The optimal conditions for maximum lentiviral transduction with minimal cell death (Additional file 1: Figure S6A,B) was first established using a CMV-GFP lentiviral construct, showing no influence on the ability of hBM-MPCs to produce a GAG-rich matrix, as highlighted by Alcian blue staining (Fig. 6a). Expression of chondrogenic marker genes in the lentiviral transduced micromasses, as quantified by qRT-PCR, was also not affected (Additional file 1: Figure S4).

Next, hBM-MPCs were transduced with the lentiviral *COL2A1*-GFP reporter construct, placed into gelMA overlay micromass cultures, and induced to undergo chondrogenesis over a 14-day period with CM supplemented with TGFβ3. Transduced cells were mixed with un-transduced at a ratio of 1:100, with the transduced cells, thus serving as sentinel cells for the behavior of the micromass culture. The activation of the *COL2A1* promoter in the micromass culture as a function of chondrogenic differentiation was monitored as expression of the GFP reporter documented by epifluorescence microscopy (Fig. 6b). The planar morphology of the cultures under the gelMA overlay permitted the counting of individual fluorescent cells and quantitation of the fluorescence intensity of the cells in a non-invasive manner throughout the entire culture period. In contrast, in non-gelMA-covered micromass cultures, it was impossible to identify individual, fluorescent sentinel cells (Additional file 1: Figure S6C). For validation of this non-invasive assay, we also collected the cultures after fluorescence examination on days 7, 10, and 14 for qRT-PCR analysis of expression of chondrogenesis-associated genes. Comparison of the corrected total cell fluorescence (CTCF) [47, 48] generated by the *COL2A1*-GFP reporter construct and the fold changes in *COL2A1* gene expression as detected by qRT-PCR revealed an excellent correlation between the two means of detection, maintaining the same ratio at the different time points (Fig. 6c). While the number of fluorescent cells remained constant during culture period, confirming the results of the MTS assay, the fluorescence intensity of the cells increased from day 7 to day 14, indicating enhanced chondrogenic activity of the cells, consistent with the increase in glucose consumption (Fig. 2c).

## Discussion

High-density micromass cultures of embryonic limb bud mesenchymal cells have been extensively exploited to study the early events of skeletal development and are one of several in vitro systems validated for some aspects of toxicity testing. However, the high cell number requirement of the culture system and the multiple end-point analyses render the application of these cultures in high throughput experiments complicated and impractical. Culture models that are appropriate for high-throughput analysis of chondrogenesis need to be uniform, reproducible, high-content, microscalar, and comprised of a biologically relevant cell type. hBM-MPCs are generally considered as one of the most suitable cell sources for musculoskeletal tissue engineering, capable of differentiation into a wide variety of mesenchymal lineages including bone, cartilage, fat, muscle, and tendon, among others. Most importantly, hBM-MPCs have been shown to exhibit normal developmental characteristics during tissue repair and regeneration, in an autonomous, self-organized fashion. Thus, they have been used as candidate cells for functional tissue engineering, for example, in the development of functional bone and osteochondral tissues. These developmental engineering approaches usually exploit the use of an hBM-MPC-derived cartilage intermediate, reminiscent to that seen in embryonic skeletogenesis. In this study, we have generated this cartilage intermediate through a modified micromass culture of hBM-MPCs using 40,000 cells, a substantial reduction of the conventional 2 to 4 × 10^7^ cells per culture [22, 49, 50]. Specifically, in an attempt to limit usage to only cells that have not shown any reduction in ability to undergo multi-lineage differentiation, cells were used after less than 3 passages or ~ 8–9 population doubling. However, in order to maintain high cell density, this also resulted in reduction of the volume of cell suspension seeded into each culture well, causing some unexpected challenges, including compromised cell-substrate adhesion and irregular culture morphology, which we were able to overcome through physical changes of the culture substrate surface and, importantly, the use of a gelMA overlay. The encapsulation of cells with gelMA hydrogels is known to deliver and/or retain cells in a construct and direct their differentiation towards a chondrogenic phenotype [40, 44]. The application of gelMA on top of the culture as a means to help the culture retains a uniform, planar morphology of the high-density micromass culture and results in upregulated expression of chondrogenic genes, possibly related to the supplementary ECM cues presented to the cells [44].

To validate the modified gelMA overlay hBM-MPC micromass system, we characterized the cytoviability and chondrogenic differentiation profile of the system biochemically, histologically, and in terms of expression of chondrogenic genes. Cell viability and cytotoxicity tests revealed excellent viability of the hBM-MPCs under the gelMA overlay. Results from the MTS assay indicated maintenance of consistent cell number coupled with increasing glucose consumption as the culture differentiated. Glucose consumption based on changes in medium glucose concentration is a crucial piece of information both as a non-destructive measure of culture activity and differentiation, but also as a common metabolic parameter in functionally coupling the chondrogenic organotypic culture model developed here with other tissue models (see below).

Chondrogenic differentiation of micromass cultures of embryonic limb bud mesenchymal cells, isolated from chick, mice, and rats, generally shows a peculiar self-organizing pattern of evenly spaced cartilage nodules [22, 24, 51]. The morphology of such cultures is highly informative (i.e., high-content), since the degree of chondrogenesis can be easily determined by counting the number and measuring the size of the nodules, assessing the different morphologies of cells comprising the nodules, and quantifying the staining intensity of nodules, e.g., using Alcian blue. On the other hand, hBM-MPC micromass cultures induced with TGFβ3 to undergo chondrogenesis reveal uniform and simultaneous differentiation and do not yield the information that can be gathered morphometrically from embryonic mesenchymal cells. In our hBM-MPC micromass system, histology, SEM, IHC, and gene expression analyses showed time-dependent increase of chondrogenic differentiation, in terms of GAG and matrix production, collagen deposition, and fibrils assembling, as well as collagen type II and aggrecan expression at the mRNA and protein levels. Although end-point analyses such as real-time PCR, histology, and IHC are universally accepted as tools to assess the level of chondrogenesis in micromass cultures, they are not compatible with high throughput studies. In this study, we have generated hBM-MPCs transduced with a lentiviral *COL2A1* promoter-GFP reporter construct and applied them as sentinel cells in hBM-MPC micromass cultures for real-time monitoring of chondrogenesis in the system. The use of plasmid and lentiviral reporters has been used to study hBM-MPC differentiation into osteogenic, chondrogenic, and adipogenic lineages [39, 52], as well as myogenic differentiation [53]. However, these studies have been conducted on standard 2-dimensional cell culture settings, which are known to lack some of the fundamental interactions occurring during condensation of the mesenchymal cells and subsequent chondrogenesis. Tri-dimensionality and cell-cell interactions are present in a micromass setting, as shown by the increased production of tight junctions and focal adhesions [53]. Moreover, the quantitative values obtained by fluorescence analysis were not directly correlated to the traditional and established methods mentioned above. In this study, we directly correlated the value of fluorescence intensity to the gene expression fold change obtained by qRT-PCR analysis, showing parallel, time-dependent trends of the two different outputs. Although the use of a higher number of time points would certainly increase the resolution of the measurements and the correlation, our findings represent an important first step in the transition from destructive end-point analyses to non-invasive assays for faster, time-resolved readouts of the studied phenomena.

A major application of the in vitro organotypic culture model developed here is embryotoxicity testing, specifically directed to skeletogenesis. In current practice, the testing of a high number of chemicals will require a large number of laboratory animals, so using in vitro systems as pre-screens or as validated alternatives is certainly beneficial to reduce the number of whole animals used. This would decrease the costs associated with drug-development and toxicity testing. The number of analyzed genes should be certainly increased for a more robust dataset. In a developmental context, it is also important to consider that virtually all metabolites received by the fetus are produced by maternal organs [54]. Thus, to evaluate the influence of these metabolites on an in vitro embryonic tissue model, it would be ideal to enable functional connection of different human tissues in a microfluidic chip setting and to demonstrate organ-specific processing of selected compounds consistent with clinical data [53]. To this end, we are currently designing a microfluidics chip to harbor the chondrogenic hBM-MPC micromass model and functionally connect it with other tissues, e.g., liver. Such a microfluidic platform will require continuous medium flow, optical access for real-time monitoring, and multi-chamber design to allocate different cell types.

The effectiveness of this method as a tool in predicting environmental chemical toxicity is highly dependent on the MPC population employed. The MPCs used in this study are derived from patients undergoing total hip arthroplasty as a result of osteoarthritis degeneration. It has been demonstrated that the epigenetic code of MPCs differs widely based on the donor health as well as isolation, storage, expansion, and differentiation protocols employed [55, 56]. The epigenetic code and physiological state (with respect to proliferation, differentiation, and senescence) of the MPCs will have a great impact on the chondrogenic differentiation of the cultures [42, 56]. Strict adherence to a standard of operating procedures will have to be followed with respect to harvest, storage, expansion, and differentiation to maximize the predictive value of these cultures. Importantly, this study investigated the use of a non-invasive COL2 promoter-reporter construct to assess MPC chondrogenesis. This tool combined with the small size of this culture make the system presented an ideal starting point for the assessment of MPC biology and utility in cell-based regenerative techniques for cartilage and bone engineering. While we used a pooled population of cells in this study to validate the system, we envision employing this culture and method on cells derived from individual donors reflecting patient diversity and potentially replicating a population study, ultimately a strength of the system. It remains to be seen whether adult human MPCs retain real or surrogate activities that reflect embryonic and fetal skeletogenesis may be used in reproductive toxicological studies.

## Conclusion

Using in vitro models of chondrogenesis to predict human toxicity using animal cells has always been difficult due to the physiological differences between animals, such as rats, and humans. These differences are born out in the poor congruence in outcomes between controlled laboratory experiments using in vitro animal models and human clinical trials. The embryonic rat limb bud mesenchyme assay for chondrogenesis represents a relatively successful predictor of human outcomes, with a reported 86% success rate in the prediction of human toxicity; however, due to important failures, this system is not an accepted method for toxicity testing. The failure of this and other animal-based in vitro models and human-based monocultures has prompted us to develop a human cell-based micromass system. In this report, we have successfully adapted the high-density chondrogenic micromass culture system for use with adult hBM-MPCs that is amenable to high throughput culture and analysis, by providing chondrogenic signals and reducing the required cell number while retaining a reproducible planar morphology using a photocrosslinked hydrogel overlay. These miniaturized micromass cultures, when enhanced with the incorporation of lentivirally transduced fluorescent/chemiluminescent promoter-reporters for cell viability, signaling pathways and tissue-specific products will generate high throughput, high content cultures capable of studying mechanisms of human chondrogenesis and detecting pharmacologic and environmental toxicity in a rapid and cost-effective manner. We conclude that the novel hBM-MPC micromass culture described here is a reproducible and controlled organotypic culture model for the study of the chondrogenic phase of human skeletal development.

## Supplementary information

**Additional file 1:** **Figure S1.** 7 day CFU at p2 for patient 39M022515. Colonies are stained with Crystal Violet. **Figure S2.** 21-day, tri-lineage differentiation at p2 for patient 39M022515 performed in duplicate. Chondrogenesis (Alcian Blue); Osteogenesis (Alizarin Red) and Adipogenesis (Oil Red O). **Figure S3.** Surface marker profile of pooled hBM-MPC populations (8 patients) by flow cytometry for positive (CD73, CD90, CD105) and negative (CD31, CD34, CD45) markers of hBM-MPC s. **Figure S4.** Alcian blue staining of day 14 micromass cultures using different plate treatments. **Figure S5.** Molecular characterization of the micromass culture to assess the effect of gelMA overlay and lenti-viral transduction on chondrogenesis. **Figure S6.** Characterization of the micromass culture for lenti-viral transduction optimization and effect on non-invasive monitoring. **Table S1.** CFU and Differentiation Score for hBM-MPCs derived from 8 patients. **Supplementary Methods**. hMB-MPC isolation, culture and characterization.

## Data Availability

All data generated or analyzed during this study are included in this published article and its supplementary information files.

## Abbreviations

CM: Chondrogenic medium
CTCF: Corrected total cell fluorescence
ECM: Extracellular matrix
hBM-MPC: Human bone marrow-derived mesenchymal progenitor cell
gelMA: Methacrylated gelatin
GAG: Glycosaminoglycan
GM: Growth medium
*COL2A1*: Collagen type II (gene)
*ACAN*: Aggrecan (gene)

## References

[1] Canfield MA, Honein MA, Yuskiv N, Xing J, Mai CT, Collins JS (2006). National estimates and race/ethnic-specific variation of selected birth defects in the United States, 1999–2001. Birth Defects Res Part A Clin Mol Teratol.

[2] Parker SE, Mai CT, Canfield MA, Rickard R, Wang Y, Meyer RE (2010). Updated national birth prevalence estimates for selected birth defects in the United States, 2004-2006. Birth Defects Res Part A Clin Mol Teratol..

[3] Lemacks J, Fowles K, Mateus A, Thomas K (2013). Insights from parents about caring for a child with birth defects. Int J Environ Res Public Health.

[4] Christianson A, Howson CP, Modell B (2008). March of Dimes: global report of birth defects: the hidden toll of dying and disabled children.

[5] Lobo I, Zhaurova K (2008). Birth defects: causes and statistics. Nat Educ.

[6] Webb E, Bushkin-Bedient S, Cheng A, Kassotis CD, Balise V, Nagel SC (2014). Developmental and reproductive effects of chemicals associated with unconventional oil and natural gas operations. Rev Environ Health.

[7] Weinhold B (2009). Environmental factors in birth defects: what we need to know. Environ Health Perspect.

[8] Dolk H, Vrijheid M (2003). The impact of environmental pollution on congenital anomalies. Br Med Bull.

[9] Tyl R, Marr M, Taylor and Francis Group (2012). Developmental toxicity testing – methodology. Dev Reprod Toxicoogy, A Pract Approach.

[10] Esch MB, King TL, Shuler ML (2011). The role of body-on-a-chip devices in drug and toxicity studies. Annu Rev Biomed Eng.

[11] DiMasi JA, Hansen RW, Grabowski HG (2003). The price of innovation: new estimates of drug development costs. J Health Econ.

[12] Olson H, Betton G, Robinson D, Thomas K, Monro A, Kolaja G (2000). Concordance of the toxicity of pharmaceuticals in humans and in animals. Regul Toxicol Pharmacol.

[13] Basketter DA, Clewell H, Kimber I, Rossi A, Blaauboer B, Burrier R (2012). A roadmap for the development of alternative (non-animal) methods for systemic toxicity testing - t4 report*. ALTEX..

[14] DeLise AM, Stringa E, Woodward WA, Mello MA, Tuan RS (2000). Embryonic limb mesenchyme micromass culture as an in vitro model for chondrogenesis and cartilage maturation. Methods Mol Bio.

[15] Paulsen DF, Solursh M (1988). Microtiter micromass cultures of limb-bud mesenchymal cells. In Vitro Cell Dev Biol.

[16] Piersma A (2004). Validation of alternative methods for developmental toxicity testing. Toxicol Lett.

[17] Oberlender SA, Tuan RS (1994). Spatiotemporal profile of N-cadherin expression in the developing limb mesenchyme. Cell Adhes Commun.

[18] LeClair EE, Bonfiglio L, Tuan RS (1999). Expression of the paired-box genesPax-1 andPax-9 in limb skeleton development. Dev Dyn.

[19] Delise AM, Tuan RS (2002). Analysis of N-cadherin function in limb mesenchymal chondrogenesis in vitro. Dev Dyn.

[20] Ahrens PB, Solursh M, Reiter RS (1977). Stage-related capacity for limb chondrogenesis in cell culture. Dev Biol.

[21] Daumer KM, Tufan AC, Tuan RS (2004). Long-term in vitro analysis of limb cartilage development: involvement of Wnt signaling. J Cell Biochem.

[22] Mello MA, Tuan RS (1999). High density micromass cultures of embryonic limb bud mesenchymal cells: an in vitro model of endochondral skeletal development. Vitr Cell Dev Biol - Anim.

[23] Genschow E, Spielmann H, Scholz G, Seiler A, Brown N, Piersma A (2002). The ECVAM international validation study on in vitro embryotoxicity tests: results of the definitive phase and evaluation of prediction models. European Centre for the Validation of Alternative Methods. Altern Lab Anim.

[24] Spielmann H, Genschow E, Brown NA, Piersma AH, Verhoef A, Spanjersberg MQI (2004). Validation of the rat limb bud micromass test in the international ECVAM validation study on three in vitro embryotoxicity tests. Altern Lab Anim.

[25] Marx U, Andersson TB, Bahinski A, Beilmann M, Beken S, Cassee FR (2016). Biology-inspired microphysiological system approaches to solve the prediction dilemma of substance testing. ALTEX..

[26] Caplan AI, Hariri R (2015). Body management: mesenchymal stem cells control the internal regenerator. Stem Cells Transl Med.

[27] Jackson WM, Nesti LJ, Tuan RS (2012). Concise review: clinical translation of wound healing therapies based on mesenchymal stem cells. Stem Cells Transl Med.

[28] Steinert AF, Rackwitz L, Gilbert F, Nöth U, Tuan RS (2012). Concise Review: The clinical application of mesenchymal stem cells for musculoskeletal regeneration: current status and perspectives. Stem Cells Transl Med.

[29] Kolf CM, Cho E, Tuan RS (2007). Mesenchymal stromal cells. Biology of adult mesenchymal stem cells: regulation of niche, self-renewal and differentiation. Arthritis Res Ther..

[30] Tuan RS, Boland G, Tuli R (2003). Adult mesenchymal stem cells and cell-based tissue engineering. Arthritis Res Ther.

[31] Dominici M, Le Blanc K, Mueller I, Slaper-Cortenbach I, Marini F, Krause D (2006). Minimal criteria for defining multipotent mesenchymal stromal cells. The International Society for Cellular Therapy position statement. Cytotherapy..

[32] Goldberg A, Mitchell K, Soans J, Kim L, Zaidi R (2017). The use of mesenchymal stem cells for cartilage repair and regeneration: a systematic review. J Orthop Surg Res.

[33] Squillaro T, Peluso G, Galderisi U (2016). Clinical trials with mesenchymal stem cells: an update. Cell Transplant.

[34] Occhetta P, Pigeot S, Rasponi M, Dasen B, Mehrkens A, Ullrich T (2018). Developmentally inspired programming of adult human mesenchymal stromal cells toward stable chondrogenesis. Proc Natl Acad Sci U S A.

[35] Scotti C, Tonnarelli B, Papadimitropoulos A, Scherberich A, Schaeren S, Schauerte A (2010). Recapitulation of endochondral bone formation using human adult mesenchymal stem cells as a paradigm for developmental engineering. Proc Natl Acad Sci U S A.

[36] Van Den Bulcke AI, Bogdanov B, De Rooze N, Schacht EH, Cornelissen M, Berghmans H (2000). Structural and rheological properties of methacrylamide modified gelatin hydrogels. Biomacromolecules..

[37] Fairbanks BD, Schwartz MP, Bowman CN, Anseth KS (2009). Photoinitiated polymerization of PEG-diacrylate with lithium phenyl-2,4,6-trimethylbenzoylphosphinate: polymerization rate and cytocompatibility. Biomaterials..

[38] Caterson EJ, Nesti LJ, Danielson KG, Tuan RS (2002). Human marrow-derived mesenchymal progenitor cells. Mol Biotechnol.

[39] Song L, Tuan RS (2004). Transdifferentiation potential of human mesenchymal stem cells derived from bone marrow. FASEB J.

[40] Lin H, Zhang D, Alexander PG, Yang G, Tan J, Cheng AW-M (2013). Application of visible light-based projection stereolithography for live cell-scaffold fabrication with designed architecture. Biomaterials..

[41] Klumpers DD, Mooney DJ, Smit TH (2015). From skeletal development to tissue engineering: lessons from the micromass assay. Tissue Eng Part B Rev.

[42] Jiang T, Xu G, Wang Q, Yang L, Zheng L, Zhao J (2017). In vitro expansion impaired the stemness of early passage mesenchymal stem cells for treatment of cartilage defects. Cell Death Dis.

[43] Somaiah C, Kumar A, Mawrie D, Sharma A, Patil SD, Bhattacharyya J (2015). Collagen promotes higher adhesion, survival and proliferation of mesenchymal stem cells. PLoS One.

[44] Schuurman W, Levett PA, Pot MW, van Weeren PR, Dhert WJA, Hutmacher DW (2013). Gelatin-methacrylamide hydrogels as potential biomaterials for fabrication of tissue-engineered cartilage constructs. Macromol Biosci.

[45] Dexheimer V, Frank S, Richter W (2012). Proliferation as a requirement for in vitro chondrogenesis of human mesenchymal stem cells. Stem Cells Dev.

[46] Pattappa G, Heywood HK, de Bruijn JD, Lee DA (2011). The metabolism of human mesenchymal stem cells during proliferation and differentiation. J Cell Physiol.

[47] Burgess A, Vigneron S, Brioudes E, Labbé J-C, Lorca T, Castro A (2010). Loss of human Greatwall results in G2 arrest and multiple mitotic defects due to deregulation of the cyclin B-Cdc2/PP2A balance. Proc Natl Acad Sci.

[48] McCloy RA, Rogers S, Caldon CE, Lorca T, Castro A, Burgess A (2014). Partial inhibition of Cdk1 in G 2 phase overrides the SAC and decouples mitotic events. Cell Cycle.

[49] Greco KV, Iqbal AJ, Rattazzi L, Nalesso G, Moradi-Bidhendi N, Moore AR (2011). High density micromass cultures of a human chondrocyte cell line: a reliable assay system to reveal the modulatory functions of pharmacological agents. Biochem Pharmacol.

[50] Zhang L, Su P, Xu C, Yang J, Yu W, Huang D (2010). Chondrogenic differentiation of human mesenchymal stem cells: a comparison between micromass and pellet culture systems. Biotechnol Lett.

[51] Decker RS, Koyama E, Enomoto-Iwamoto M, Maye P, Rowe D, Zhu S (2014). Mouse limb skeletal growth and synovial joint development are coordinately enhanced by Kartogenin. Dev Biol.

[52] Padmashali RM, Mistriotis P, Liang M, Andreadis ST (2014). Lentiviral arrays for live-cell dynamic monitoring of gene and pathway activity during stem cell differentiation. Mol Ther.

[53] Moharil J, Lei P, Tian J, Gaile DP, Andreadis ST (2015). Lentivirus live cell array for quantitative assessment of gene and pathway activation during myogenic differentiation of mesenchymal stem cells. PLoS One.

[54] Vernetti L, Gough A, Baetz N, Blutt S, Broughman JR, Brown JA (2017). Functional coupling of human microphysiology systems: intestine, liver, kidney proximal tubule, blood-brain barrier and skeletal muscle. Sci Rep.

[55] Cakouros D, Gronthos S (2019). Epigenetic regulation of bone marrow stem cell aging: revealing epigenetic signatures associated with hematopoietic and mesenchymal stem cell aging. Aging Dis.

[56] Yang Y-HK, Ogando CR, See CW, Chang T-Y, Barabino GA (2018). Changes in phenotype and differentiation potential of human mesenchymal stem cells aging in vitro. Stem Cell Res Ther.

