# Supplementary material for: Modeling appendicular skeletal cartilage development with modified high-density micromass cultures of adult human bone marrow-derived mesenchymal progenitor cells
Source: Stem Cell Res Ther. 2019 Dec 16;10:388. doi: 10.1186/s13287-019-1505-5 (PMC6916440; doi:10.1186/s13287-019-1505-5)
Supplement: Supplementary file 1 — Additional file 1: Figure S1. 7 day CFU at p2 for patient 39M022515. Colonies are stained with Crystal Violet. Figure S2. 21-day, tri-lineage differentiation at p2 for patient 39M022515 performed in duplicate. Chondrogenesis (Alcian Blue); Osteogenesis (Alizarin Red) and Adipogenesis (Oil Red O). Figure S3. Surface marker profile of pooled hBM-MPC populations (8 patients) by flow cytometry for positive (CD73, CD90, CD105) and negative (CD31, CD34, CD45) markers of hBM-MPC s. Figure S4. Alcian blue staining of day 14 micromass cultures using different plate treatments. Figure S5. Molecular characterization of the micromass culture to assess the effect of gelMA overlay and lenti-viral transduction on chondrogenesis. Figure S6. Characterization of the micromass culture for lenti-viral transduction optimization and effect on non-invasive monitoring. Table S1. CFU and Differentiation Score for hBM-MPCs derived from 8 patients. Supplementary Methods. hMB-MPC isolation, culture and characterization. [file 13287_2019_1505_MOESM1_ESM.docx]

**Additional file 1**

**Figure S1**: 7 day CFU at p2 for patient 39M022515. Colonies are stained with Crystal Violet.

**Figure S2**: 21-day, tri-lineage differentiation at p2 for patient 39M022515 performed in duplicate. Chondrogenesis (Alcian Blue); Osteogenesis (Alizarin Red) and Adipogenesis (Oil Red O).

**Figure S3**: Surface marker profile of pooled hBM-MPC populations (8 patients) by flow cytometry for positive (CD73, CD90, CD105) and negative (CD31, CD34, CD45) markers of hBM-MPC s.

**Figure S4**: Alcian blue staining of day 14 micromass cultures using different plate treatments. C- (no etching, no PureCol treatment) and Etched showed irregular morphology and contraction of the micromass, PureCol treatment improved morphology but a large number of cells crawled out of the micromass, whereas both sand-paper etching and PureCol coating gave best results in terms of cell attachment and morphology.

**Figure S5**: Molecular characterization of the micromass culture to assess the effect of gelMA overlay and lenti-viral transduction on chondrogenesis. Gene expression analysis measured by qRT-PCR showed higher levels of the chondrogenic genes (A) *COL2A1* and (B) *ACAN.* Results are fold changes of gene expression in the presence of gelMA overlay compared to no-gelMA cultures. No differences were seen between transduced and untransduced micromasses.

**Figure S6**: Characterization of the micromass culture for lenti-viral transduction optimization and effect on non-invasive monitoring. (A) MOI = 50 and SureENTRY = 6 μg/ml (data not shown) showed best transduction efficiency. Scale bar = 100 μm. (B) Lenti-viral transduction and SureENTRY reagent did not affect cell viability measured by MTS assay. (C) Micromass cultures not covered with gelMA overlay were not amenable to non-invasive analysis due to the inability to identify individual sentinel cells. Scale bar = 1 mm.

| **Table S1. CFU and Differentiation Score for** **hBM-MPCs derived from 8 patients** | | | | | | | | | | | |
| --- | --- | --- | --- | --- | --- | --- | --- | --- | --- | --- | --- |
| Patient ID | Staining | | | #CFU (P0) (per 10 million cells) | | | | #CFU (P2) (per 100 cells) | | | |
|  | Chondro | Osteo | Adipo | Dish 1 | Dish 2 | Dish 3 | Avg | Dish 1 | Dish 2 | Dish 3 | Avg |
| 56M041510 | 2+ | 3+ | 1+ | / | / | / | / | 16 | 16 | 19 | 17 |
| 38F041112 | 2~3+ | 2+ | 1+ | too many to count | | | / | 22 | 31 | 27 | 27 |
| 60F041812 | 2+ | 3+ | 1+ | 45 | 46 | 52 | 48 | 21 | 23 | 20 | 21 |
| 53F092612 | 2+ | 2+ | 2~3+ | 101 | 99 | 77 | 92 | 44 | 38 | 40 | 41 |
| 76F071013 | 2+ | 2+ | 2+ | 60 | 77 | 72 | 70 | 17 | 23 | 19 | 20 |
| 55M111413 | 2+ | 3+ | 2+ | 6 | / | 9 | 8 | 43 | 49 | 44 | 45 |
| 59M030514 | 2~3+ | 2+ | 3+ | 50 | 39 | 46 | 45 | / | / | / | / |
| 39M022515 | 3+ | 2~3+ | 3+ | 27 | 28 | 27 | 27 | 52 | 44 | 53 | 50 |


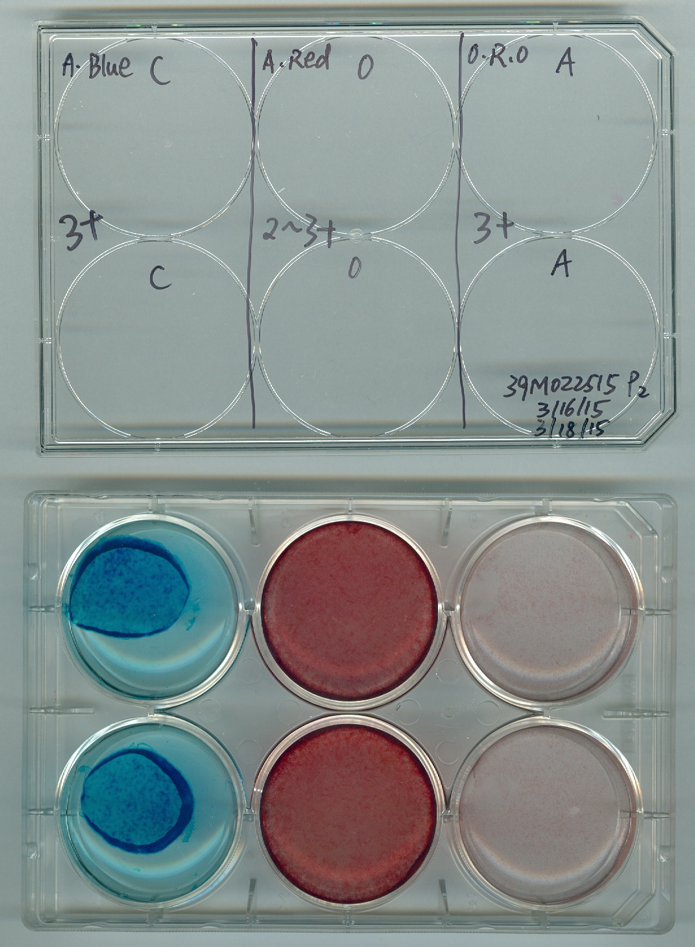


Figure S2

**Chondrogenesis**

**Osteogenesis**

**Adipogenesis**


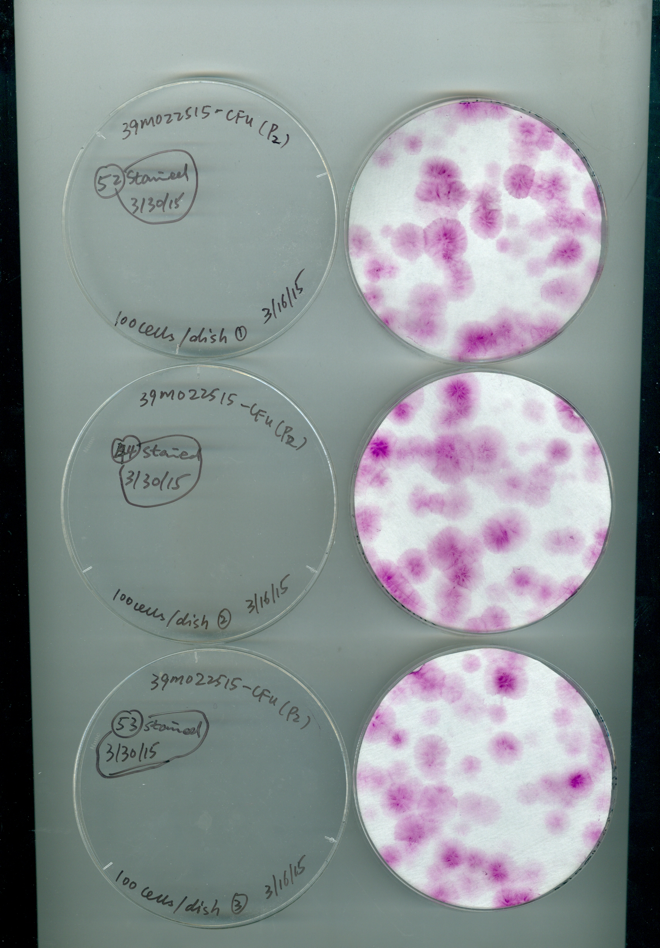


Figure S1


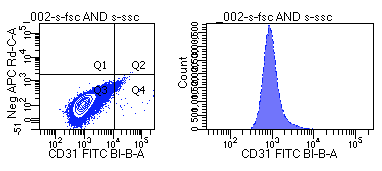

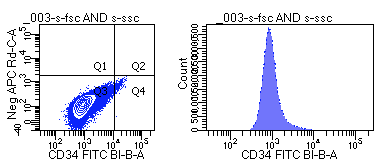

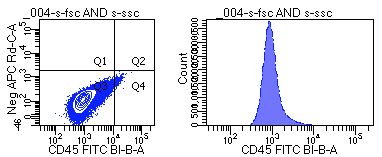

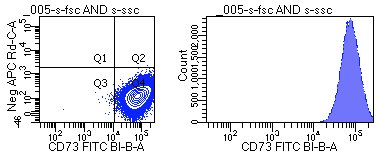

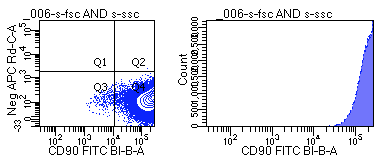

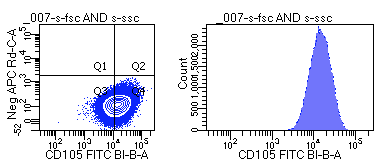


**Positive Markers for hBM-MPCs**

**Negative Markers for hBM-MPCs**

**CD73**

**CD90**

**CD105**

**CD31**

**CD34**

**CD45**

Figure S3


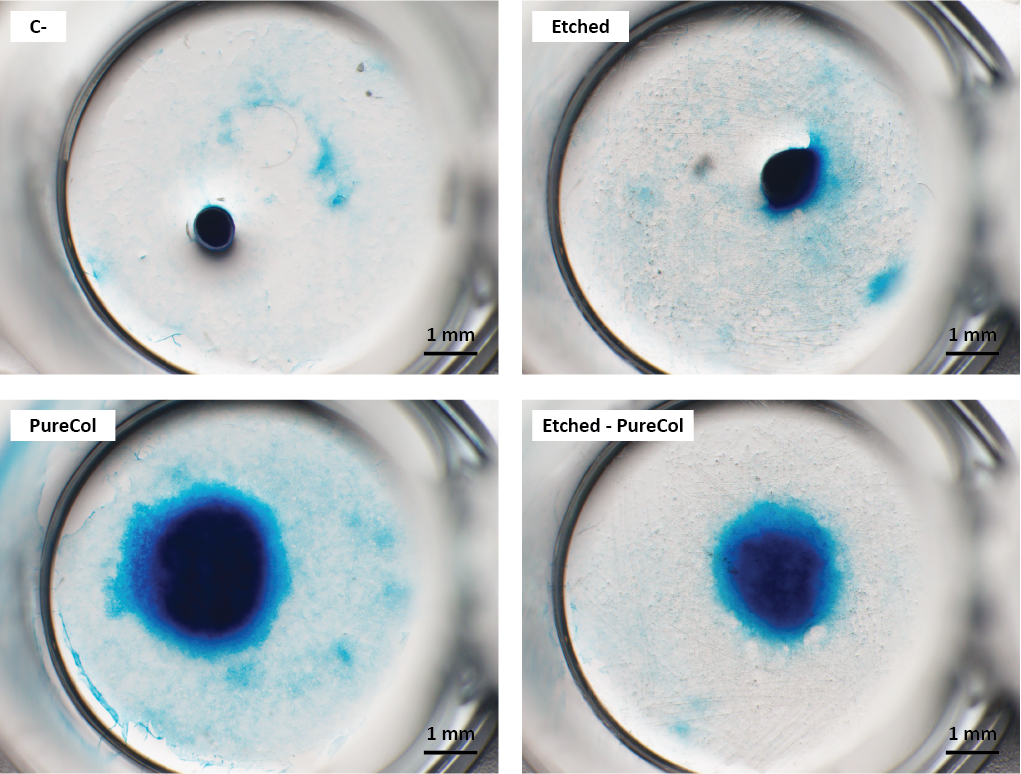


Figure S4

Figure S5


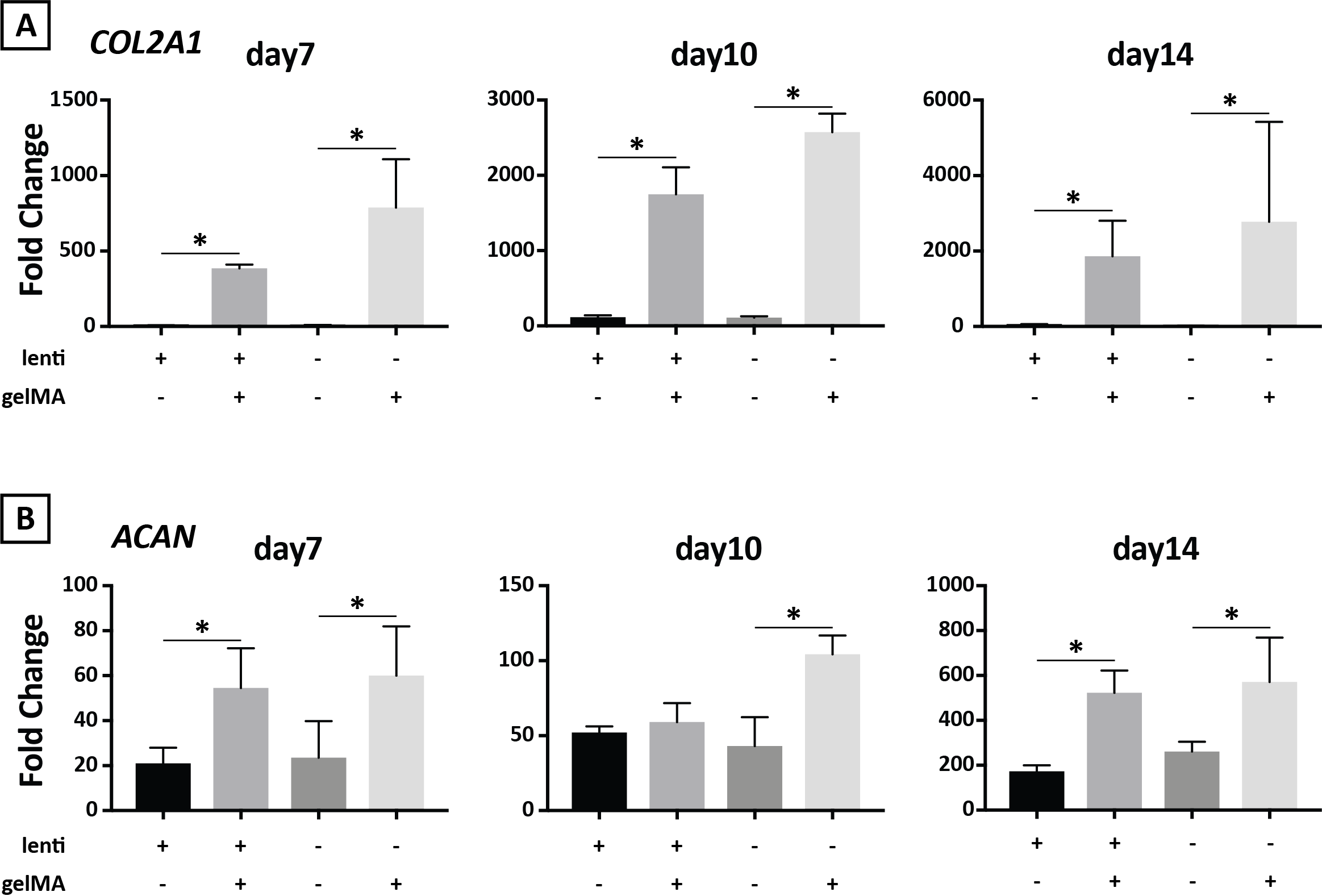


**Supplementary Methods:**

Figure S6


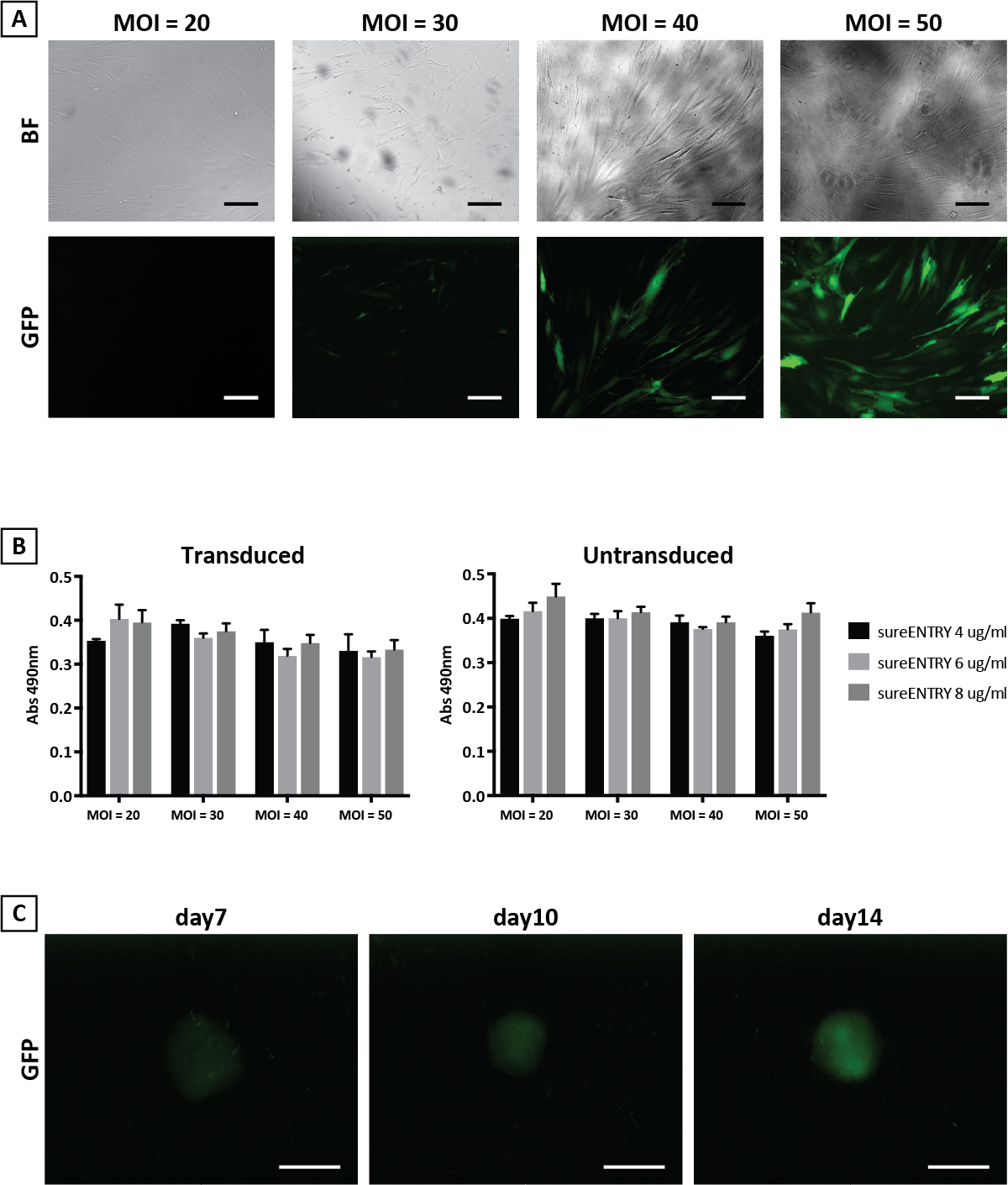


hBM-MPC isolation and expansion: Bone marrow was obtained from the femoral heads patients undergoing total hip arthroplasty with the approval of the institutional review board of University of Pittsburgh. Aspirated marrow was placed into 50mL conical tubes and supplemented with Basal Medium (alpha MEM, 10% FBS, 1x antibiotic/antimycotic (consisting of 100 units/ml penicillin, 100 μg/ml streptomycin, 25 ng/ml Fungizone) and centrifuged at 230xg for 5 min. The marrow separated into an upper serous layer and a lower densely packed red blood cell layer. The serous layer was aspirated from the red blood cell layer and the cell layer was resuspended in anther 50ml of medium and washed as above. The remaining pale pink cell pellet (bone marrow stromal fraction) was resuspended in 1 ml BM, a small aliquot removed for CFU analysis and the remaining cells resuspended in 25 ml of BM supplemented with 2 ng/ml FGF2 and plated in a T150. Nonadherent cells were removed from cultures after 24 h by a series of phosphate buffered saline (PBS) washes and subsequent medium changes. Adherent cell clusters gave rise to colony forming fibroblastic cells and were expanded as monolayer cultures in a 5% CO2 atmosphere at 37ºC with medium changes every three days for periods of up to three weeks prior to experimentation. All cells, now referred to as human mesenchymal stem cells (hBM-MPCs), used in the studies presented here were used at passage 3 (p3). Cells displayed an undifferentiated phenotype, i.e., fibroblastic in shape, small size, absence of cytoplasmic inclusions, and lacking in significant metachromatic extracellular matrix deposition.

CFU and crystal violet staining: After final centrifugation (before plating) or before plating trypsinized cells at p3, total nuclear cell counts were determined by hemocytometer. At p0, cell suspensions were diluted to 1 x 10^6^ cells/ml and at p2 trypsinized cells were diluated to 100 cells/ml and 1 ml of the diluted cell suspensions were added to 10 ml of BM in a 100 mm petri dish. The cultures was maintained using BM in a humidified, 37°C incubator for 7 days with medium changes every 2-3 days. After 7 days, cultures were washed with PBS, fixed in 10% formalin for 15 minutes, rinsed with distilled H2O, stained with 0.01% (w/v) crystal violet in dH2O for 30-60 minutes, washed with distilled H2O and dried on the benchtop at RT°C. Colonies were counted by an experienced invitrologist (JT).

Differentiation: (1) Chondrogenic differentiation was performed on hBM-MCPs in monolayer cells at 10^4^ cells/cm^2^ in serum-free chondrogenic medium (DMEM containing 0.1 μM dexamethasone, 50 μg/ml L-ascorbate 2-phosphate, 40 μg/ml L-proline, 100 μg/ml sodium pyruvate, ITS-Plus (BD Biosciences, Bedford, MA), and 10 ng/ml TGF-β3 (R and D Systems, Inc., Minneapolis, MN) for 21 days. (2) To induce osteogenesis, hBM-MPCs were cultured at 10^4^ cells/cm^2^ in osteogenic medium (OM; BM supplemeted with 10 nM dexamethasone, 10 mM β-glycerophosphate, 50 μg/ml L-ascorbate 2-phosphate, and 10 nM 1α,25-dihydroxyvitamin D3 for 21 days. (3) Adipogenic differentiation was induced at a cell density of 104 cells/cm2 in adipogenic medium (AM; BM supplemented 10 μMdexamethasone, 1 μg/ml insulin, and 0.5 mM 3-isobutyl-1- methylxantine (IBMX)) for 21 days. Media changes were done every 2-3 days for all cultures.

Staining: (1) For Alcian Blue staining, cultures were fixed in 70% ethanol for 15 minutes, rinsed with distilled water, incubated in 3% acetic acid (in H2O) for 15 minutes before staining with Alcian Blue (Cat# 1198, EK Industries) at pH = 1.0 overnight at 4C, repeated washing with acid acid alcohol (1% HCl in 100% ethanol) until rinse is clear, tinsing with distilled water and visualization and imaging. (2) For Alizarin Red staining, cultures were fixed in 70% ethanol for 15 minutes, rinsed with tap water before staining with Alizarin Red (Alizarin Red S Solution, 2%, pH4.2 (Cat# C-206, Rowley Biochemical Institute)) for 30 min on a a shaker at RTC, subsequent washing with tap water for 5 minutes each until the rinse is clear, and observation and imaging. (3) For Oil Red O staining, 10% formalin for 15min, washed 3x in distiolled in dH2O before stainin in Oli Red O in H2O (Oil Red O Solution Solution J-609-1, Rowley Biochemical Institute) for 2 hours, exhaustive rinsing in distilled water before observation and imaging.

Staining was qualitatively assessed by an experienced invitrologist (JT) on a scale of 0-3 (0 = no staining to 3 = strong staining).
